# Manual Training in Schools

**Published:** 1890-10-04

**Authors:** 


### Manual Training in Schools.

### I.—Its Use and Abuse.

The education of the young, as we all know, has of late been advancing by leaps and bounds, not merely in its accomplished facts, but in the keen interest which it arouses in the minds of all thoughtful, and indeed, all practical men, whether in our own country, on the Continent, or in America. Suggestions are being made, experiments tried—and very costly experiments some of them are too; new subjects and new methods of teaching are discussed, some of them, no doubt, to be finally dropped, with or without a trial, others destined, as we cannot but believe, to do a great work among the rising generation. And first and foremost among these is, we should say, manual training.

It is very generally felt that the education given in our primary schools has not been entirely satisfactory in its results. There has been too much book-learning, too much dependence on the teachers; too much putting into, and too little drawing out of, the child; too much theory, too little practice. The bad results arising from this defective method of teaching are manifold. For one thing, a child who has never been taught to think for itself, or to apply its knowledge, must be pronounced deficient in intellectual culture. Again, it is manifestly unfit to make its way in the world, being deficient in practical knowledge and power. Once more, exclusive attention to books and intellectual work tends to give the impression that manual labour is degrading, and to create, at least among the brighter and cleverer children, a positive distaste for it. Thus to divorce brains from labour is a serious evil; and that the evil is a real one may be seen by the appalling number of youths who compete for clerkships, bringing down salaries almost to starvation point, yet deliberately choosing to lead a monotonous and poverty-stricken life, so long as they can but wear a black coat, and keep themselves unspotted by manual labour. Much the same thing occurs in Germany. "To-day," says Herr Von Schenckendorff, of Görlitz, "the child leaving the school starts in life with almost a dread of manual work. . . . Thus is originated a conflicc between inclination and necessity, nourishing social discontent."

Those who would introduce manual training into our schools, then, advocate it on three grounds. Firstly, it is part and parcel of true education, being in fact not only manual, but also mind training. Secondly, it prepares a child for the practical work of after-life. Thirdly, it upholds the dignity of manual labour.

The relative importance of the first and second of these considerations will be differently estimated by different minds. Those who regard education merely from a utilitarian point of view—the practical party, as we may call them—will naturally lay most stress upon the fact that manual training will fit our children for the business of life, enabling them to earn their own bread at an earlier age, and to compete successfully with their foreign rivals in the struggle for existence. For our own part, however, we think the "bread-and-butter argument," as it has been called, is not the one which should be most strongly insisted upon. Once admit that the end of manual training is to earn one's bread, and it will degenerate at once into mere instruction, rather than education, it being unfortunately true that, as far as immediate and tangible results are concerned, it pays much better to give a child a certain amount of mechanical skill than to show it the whys and wherefores of things, and teach it to think for itself. The fact cannot be too strongly insisted upon—that a school is not a place in which to learn mechanical skill, but "a preparation for complete living." Forget this, and manual training becomes a delusion and a snare.

As to its real significance and value, we cannot do better than quote from one or two American writers on the subject, America having taken up the question with great ardour during the last ten or twelve years.

"Even those who advocate manual training," says an educational leaflet published by the great Industrial Association of New York, "are not always clear as to what it means. They often speak of substituting manual training for mental work. This is incorrect. The substitution is of one form of mental work for another. Manual training, in the sense in which it is here used, is a training of the mind to accuracy of perception and truthfulness and readiness of expression; otherwise, it would have no proper place in the public school course. The schools are not established for the purpose of teaching pupils how to make a living, but to teach them how to live."

Professor Le Conte, of the State University of California, states the philosophy of the subject very clearly. "Psychical life," he says, "is made up of three departments—the senses, the intellect, and the will; that is, observing, thinking, and doing. Development is only possible through the co-operation of these. In natural education the three are co-ordinate, but they are not co-ordinate in the artificial education of the schoolroom, for that is at best but reading, thinking, expressing . . . . Science must be taught by new methods; observing and doing must co-operate with thinking."

Another American writer, General F. A. Walker, points out that the town boy, especially, to whom are denied the hundred opportunities for using hands and eyes, planning, thinking, and doing, of the country boy, imperatively needs to be taught by manual training, how to "make the mind itself the real master of life." And, as the testimony of those who have actually watched the working out of the new method of teaching is of special value, we select one of the many favourable and indeed enthusiastic reports by school teachers, sent in to the American Commissioner of Education.

"It is the united testimony of the teachers," says Superintendent Chapman, of New Jersey, "that the pupils who attend the industrial school retain their places or standing in their respective classes. No falling off in any particular has been noted. On the other hand, the change of work and the stimulus to excel in this particular kind of knowledge has rather added to the work the pupils are doing on their regular lessons. The fact that a dull boy has shown his class that he can do something has tended to elevate the standing of that particular boy, not only in his own estimation, but in that of his comrades."

We have already insisted, and would insist again, on the paramount importance of putting the educational value of manual training in the first place, the bread-and-butter value in the second. Still, we can by no means afford to despise the bread-and-butter argument, nor is there any reason why we should. While strongly holding that school is the place for what we may call the all-round development of the human faculties, we quite admit that special attention may be paid—must be paid—to those faculties which are specially likely to be needed. It is, in fact, impossible (one cannot but wish it were not so) for any of us, even in the course of a long life, to develop to the full our varied powers of mind and body; we must pick and choose, whether we like it or not. In children's schools, then, due regard must be paid to the life which the scholars will in future lead; and in this connection manual training may be expected to produce most valuable practical results. Only it must never aim at producing mere mechanical skill, or proficiency in any particular trade. That, if it is needed, must come later—must be taught in regular manual schools (though even here we believe the true method to be—not to teach a trade, but rather "to teach the principles underlying all trades") or in apprenticeships. In elementary schools, manual training must be understood to mean, not the mere training of the hand, but the education of the brain through the hand, and of the hand through the brain; then, and then alone, can it do all that is expected of it, alike from an educational and from a practical point of view.

In another article we hope to glance at what is actually being done in American and Continental schools in the way of manual training.

*(To be continued.)*

